# Does rhythmic entrainment represent a generalized mechanism for organizing computation in the brain?

**DOI:** 10.3389/fncom.2012.00085

**Published:** 2012-10-25

**Authors:** Kai J. Miller, Brett L. Foster, Christopher J. Honey

**Affiliations:** ^1^Department of Neurosurgery, Stanford UniversityStanford, CA, USA; ^2^Department of Neurology and Neurological Sciences, Stanford UniversityStanford, CA, USA; ^3^Department of Psychology and Princeton Neuroscience Institute, Princeton UniversityPrinceton, NJ, USA

Perceptions are changing concerning the functional role of rhythmic brain activity. There is increasing evidence that low-frequency (2–25 Hz) neural oscillations are not simply epiphenomena, but reflect an essential mechanism for coordinating brain function. A leading mechanistic hypothesis for how rhythms might directly influence neural circuit function is by synchronously modulating the membrane potentials of many neurons such that mutual dendritic input between members of this population is more likely to induce action potentials at specific times (Volgushev et al., 1998). This proposed pathway of influence matches the observation that, across numerous cortical sites and behaviors, single cell and population spiking is selectively enhanced at specific phases of ongoing narrow-band oscillations in the field potential (Murthy and Fetz, 1992; O'Keefe and Recce, 1993).

Given that coherent electrical fields sum spatially, the same distributed rhythmic brain process can be observed across multiple scales of measurement. The large-scale relationship between aggregate spike timing and these widespread rhythms has not been fully explored, because spiking behavior has generally been measured at the scale of the single neuron. In recent years, it has been observed that increases and decreases in local firing rate are accompanied by upward and downward broadband shifts in the power spectrum of the cortical surface potential (Miller, 2010). In human electrocorticographic (ECoG) measurements, this broadband signal component, reflecting aggregate firing rate, is modulated by the phase of narrow band brain rhythms (Miller, 2010). These broadband shifts in the ECoG power spectrum are most plainly revealed at high frequencies (e.g., 65+ Hz, the “high gamma” range) that are well above those of canonical oscillatory brain rhythms. It has been shown in a variety of behavioral settings that the amplitude (power) in this sub-range is also modulated by the phase of low-frequency rhythms—“phase amplitude coupling” (PAC) (Miller et al., 2010, 2012; Voytek et al., 2010; Foster and Parvizi, 2011; Allen et al., 2012). As such, this modulation reflects a macroscopic index of spike-field interaction, and provides evidence that spiking activity in widespread cortical circuits can be entrained with the phase of underlying rhythms.

**Figure 1:**
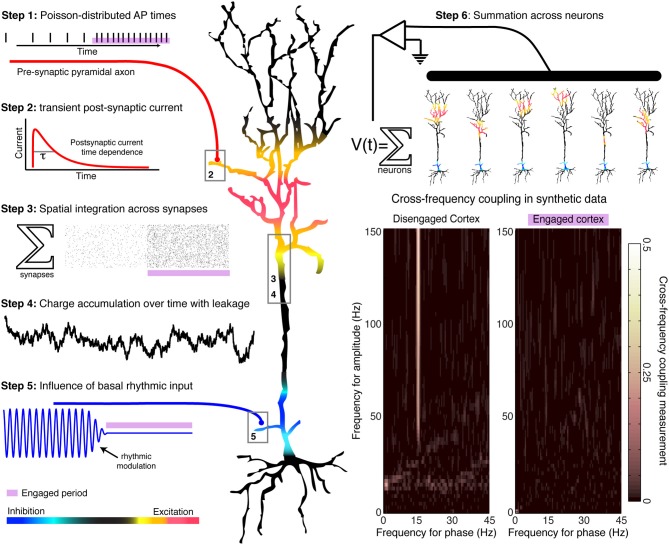
**Generation of a simulated time series, with and without rhythmic entrainment.** The illustrated heuristic was used to generate a 1/f base, broadband, synthetic time series that is entrained by a 15 Hz rhythm. The steps are numbered in the illustration: Poisson-distributed distal-dendrite synaptic inputs are each filtered by the shape of the post-synaptic current, and are integrated over space and time across the dendritic arbor. This summated input is then modulated by the frequency of proximal rhythmic inhibitory synaptic input. The timecourse of the current dipole potential is summated across many such model neurons. In accordance with experimental findings, periods of relative cortical “engagement” during task are devoid of the phase modulation of distal input, and periods of “disengagement” have phase modulation by the input rhythm. Aggregate rhythmic entrainment, revealed in the cross-frequency co-modulograms on the bottom right, shows significant entrainment during “disengagement,” and none during task “engagement.” Illustration and synthetic data construction adapted from Miller et al. (2010).

In general terms, low-frequency oscillations appear to be specialized more for homeostatic, organizational brain processes, rather than for actively controlling spatiotemporally precise local computations as they occur. One reason for this is that the 40–500 ms timescales corresponding to each full rhythmic cycle (2–25 Hz) are slower than many of the timescales of perception and action. Secondly, low frequency oscillations are coherent across centimeters of the cortical surface, and thus can exert a similar influence on local circuits that implement dramatically different computations. Thirdly, the modulation of population firing by low-frequency rhythms is usually greater during rest conditions than during perceptual or motor tasks, when rhythms are often suppressed (desynchronized)—such that rhythmic entrainment is most pronounced during disengaged cortical states (Miller, 2010). Thus, the amplitude and phase of a spatially coherent rhythm can provide a general constraint on the computational state of local circuits. When rhythms are strong, circuits exhibit more regulated spiking patterns that are likely associated with processes such as inter-regional communication or priming before computation; when rhythms are weak, then circuits are freed from a restricted periodic regime to engage in more of the specialized computations specific to local wiring. Notwithstanding these general observations, the specific role of rhythmic entrainment upon spiking activity in local circuit computations is in need of mechanistic elucidation. A simple heuristic model that reproduces experimental patterns of phase entrainment can be developed (Figure 1), based on the assumption that cortical rhythms arise from the reciprocal interaction of inhibitory and excitatory neurons (Yamawaki et al., 2008). Synaptic-level experiments will be needed to select between candidate models of the mechanisms of rhythmic entrainment.

Findings are now emerging concerning the higher order spatial and temporal properties of rhythmic entrainment as captured by PAC. In a recent article van der Meij et al. reported on the spatial distribution of PAC across human cerebral cortex (van der Meij et al., 2012). van der Meij et al. showed that inter-electrode PAC is observed not only for single canonical oscillations (e.g., theta band), but for multiple frequencies of phase ranging from 2 to 16 Hz. PAC was also observed across large distances (>10 cm). The authors posit that this diversity of phase coupling may allow for selective information routing between parallel brain networks, via frequency and phase multiplexing that uniquely tags dynamic functional processes to their constituent networks. Such findings are consistent with recent microscale observations that rhythmic oscillations in one brain area can influence the timing of individual spikes in another (Canolty et al., 2010). Therefore, by exploring the properties of local entrainment, one might then seek to establish a link with the putative functional role of long range rhythmic entrainment (Siegel et al., 2012). These findings in conjunction with similar reports from human ECoG have promoted the proposal that phasic entrainment be viewed as a dynamic modulation of excitability or gain, biasing the firing rates of neurons and, by extension, coordinating the spiking of neural circuits that share a rhythmic influence.

Support for this hypothesis often draws an analogy to the “up-down state” modulations of firing rate that are most typically seen in states of cortical suppression and during sleep. However, the contribution of PAC in dynamic brain function is difficult to assess in the absence of a clear neurophysiologic mechanism. Therefore, the claims made for the role of PAC in cognitive computation should be followed by: (i) a more clearly elucidated physiological basis for rhythmic entrainment processes and (ii) systematic and targeted studies of how PAC changes in relation to specific perceptual and behavioral events.

Interestingly, the rhythmic entrainment may itself have oscillatory nesting on much slower (infraslow) timescales. For example, in posterior-medial regions of the default-mode network, there is a slow 0.1 Hz fluctuation of the rhythmic entrainment, paralleling the hemodynamic fluctuations observed by neuroimaging from the same areas (Foster and Parvizi, 2011). Also, multiple rhythmic entrainments can exist within the same population of occipital neurons, and these may selectively dissipate (e.g., for one rhythm but not the other) on a second-by-second basis during visual task engagement (Miller, 2010). This evidence from the visual cortex suggests that the rhythm to which spiking is entrained will only rarely modulate one another. When looking across widespread cortical areas, there are rhythm-selective changes in phase entrainment, where two rhythms appear to exhibit a “see-saw” relationship between tasks. For example, the strengths of rhythmic entrainment of high-frequency power by theta and alpha rhythms are differentially altered in anterior versus posterior cortices when visual tasks are compared with non-visual tasks (Voytek et al., 2010). In light of these observations, the recent findings of van der Meij et al. supports multiple interpretations (van der Meij et al., 2012): the spatially distributed interactions between different rhythms may indeed reveal overlapping rhythmic coordination processes, but then, what happens to each rhythm's influence within the region of overlap? Is there simply incidental spatial overlap of processes that separately and independently organize distributed neural function? Or do all of the observed permutations collectively compose a higher-order cortical symphony?

The findings discussed here point to a large variety of interacting motifs within and between rhythmic phenomena in the brain, spanning many spatial and temporal scales. Constrained experiments and analyses are needed if we are to disentangle and definitively answer the questions that now present themselves. Specifically, we propose studies in which only one or two brain rhythms are empirically defined: the changes in rhythmic dynamics should then be characterized with respect to one associated behavior, and results should be segregated by anatomic boundaries for clear association between brain loci and physiology (Foster and Parvizi, 2011). The clearest opportunities are in primary cortices, where canonical rhythms (e.g., theta, alpha, and beta) are known to wax and wane across behavioral states, and where classical paradigms exist to control perception and behavior (Miller et al., 2012). Future experiments in these settings can seed a systematic and mechanistic theory of rhythmic entrainment and can help to adjudicate its role in neural computation.
